# Towards the Atlas of human African trypanosomiasis

**DOI:** 10.1186/1476-072X-8-15

**Published:** 2009-03-18

**Authors:** Giuliano Cecchi, Massimo Paone, José R Franco, Eric M Fèvre, Abdoulaye Diarra, José A Ruiz, Raffaele C Mattioli, Pere P Simarro

**Affiliations:** 1 Food and Agriculture Organization of the United Nations (FAO), Animal Production and Health Division, Viale delle Terme di Caracalla, 00153, Rome, Italy; 2 World Health Organization, Control of Neglected Tropical Diseases, Innovative and Intensified Disease Management, 1211 Geneva 27, Switzerland; 3 Centre for Infectious Diseases, University of Edinburgh, Ashworth Laboratories, Kings Buildings, West Mains Road, Edinburgh EH9 3JT, UK; 4 World Health Organization, Regional Office for Africa, Brazzaville, Congo; 5 World Health Organization, Regional Office for the Eastern Mediterranean, Cairo 11371, Egypt

## Abstract

**Background:**

Updated, accurate and comprehensive information on the distribution of human African trypanosomiasis (HAT), also known as sleeping sickness, is critically important to plan and monitor control activities. We describe input data, methodology, preliminary results and future prospects of the HAT Atlas initiative, which will allow major improvements in the understanding of the spatial distribution of the disease.

**Methods:**

Up-to-date as well as historical data collected by national sleeping sickness control programmes, non-governmental organizations and research institutes have been collated over many years by the HAT Control and Surveillance Programme of the World Health Organization. This body of information, unpublished for the most part, is now being screened, harmonized, and analysed by means of database management systems and geographical information systems (GIS). The number of new HAT cases and the number of people screened within a defined geographical entity were chosen as the key variables to map disease distribution in sub-Saharan Africa.

**Results:**

At the time of writing, over 600 epidemiological reports and files from seventeen countries were collated and included in the data repository. The reports contain information on approximately 20,000 HAT cases, associated to over 7,000 different geographical entities. The oldest epidemiological records considered so far date back to 1985, the most recent having been gathered in 2008. Data from Cameroon, Central African Republic, Chad, Congo, Equatorial Guinea and Gabon from the year 2000 onwards were fully processed and the preliminary regional map of HAT distribution is presented.

**Conclusion:**

The use of GIS tools and geo-referenced, village-level epidemiological data allow the production of maps that substantially improve on the spatial quality of previous cartographic products of similar scope. The significant differences between our preliminary outputs and earlier maps of HAT transmission areas demonstrate the strong need for this systematic approach to mapping sleeping sickness and point to the inaccuracy of any calculation of population at risk based on previous maps of HAT transmission areas. The Atlas of HAT will lay the basis for novel, evidence-based methodologies to estimate the population at risk and the burden of disease, ultimately leading to more efficient targeting of interventions. Also, the Atlas will help streamline future field data collection in those parts of Africa that still require it.

## Background

The human form of tsetse-transmitted trypanosomiasis, also known as sleeping sickness, is a disease unique to Africa that leads to death if untreated. Two different forms of the disease exist, depending on the parasite involved. *Trypanosoma brucei gambiense *is found in Western and Central Africa; it causes a chronic infection with a long asymptomatic phase, and it accounts for over 90 percent of total reported cases of human African trypanosomiasis (HAT). *T.b. rhodesiense*, responsible for the acute form, is found in Eastern and Southern Africa; this parasite species, which was proved to be transmissible from wild and domestic animals to humans [1,2] is responsible for less than 10 percent of reported cases [3]. The divide between the two forms can be roughly placed along the Great Rift Valley [4].

In the last decades of the twentieth century, lack of surveillance and funds for treatment programmes allowed HAT incidence to climb to alarming levels, which in 1997 prompted a resolution by the World Health Organization (WHO) advocating access to diagnosis and treatment and the reinforcement of surveillance and control activities [5]. To achieve the objectives of the World Health Assembly resolution, the WHO HAT Control and Surveillance Programme established a new initiative based on a global alliance of all actors concerned with the disease [6]. Non-Governmental Organizations (NGOs) and bilateral cooperation played a major role in the control of the HAT but the signing of a public-private partnership between WHO and sanofi-aventis in May 2001, renewed in October 2006, marked a turning point in sleeping sickness control. This partnership contributed to WHO's efforts to fight the disease, making it possible to distribute drugs free-of-charge. The partnership also allowed WHO to reinforce countries' capacity for screening populations at risk, providing care to individuals carrying the parasite and training personnel. Discontinuation of civil strife in countries where HAT is endemic, most notably in Angola, Democratic Republic of the Congo (DRC), and Sudan, facilitated access to diagnosis and treatment to people living in the areas of highest endemicity. Globally speaking, field control activities scaled up, thus leading to a reduction in disease transmission and to a better knowledge of sleeping sickness distribution in sub-Saharan Africa as a whole. More reliable country-level information on disease occurrence became available and it was published by WHO in 2006 [7] (later updated in 2008 [3]). In spite of some persisting information gaps, e.g. in Liberia, Nigeria and Sierra Leone, the extensive information collected during the last years has substantially reduced the uncertainties that surrounded disease figures prior to 1997.

Thirty-six sub-Saharan countries are considered endemic for one or the other form of HAT, although some of them have reported no cases in the last decade. Among endemic countries, DRC, Angola and Sudan are the most severely affected, respectively reporting an average of 12,057, 2,702 and 1,821 new cases per year during the period 2000–2007 (corresponding to 88.2 percent of the total Gambian cases reported). Sleeping sickness incidence in these three conflict-ridden countries points to the key role played by civil strife in maintaining conditions conducive to disease transmission [8].

Despite evidence of transmission in peri-urban environments [9], HAT is usually found in remote rural areas where health systems are weak or non-existent, a fact that, in itself, makes case reporting problematic [10]. It is also a highly focal disease often characterized by distinct outbreaks in a specific area or village [11]. Sleeping sickness endemic areas receive their names from geographical features such as rivers, villages or towns, and administrative divisions [12], and the size of these areas can range from a single populated place to an entire region. Within a given endemic area, the intensity of the disease can vary from one village to the next. Also, the geographical extent of foci may change significantly over time, as a result of both human mobility (e.g. expansion of the *T.b. gambiense *focus caused by civil instability on the Sudanese border [13]) and of environmental dynamics and modifications influencing tsetse fly presence, density and dispersal [14,15]. Furthermore, it was shown that the Rhodesian form of the disease may be introduced into previously unaffected areas by cattle movement [16].

A map of the distribution and extent of transmission areas based on over 250 active and historical foci in Africa was assembled for WHO in the nineties, and it was generated from formal and grey literature, as well as from expert opinion [17]. However, in 2005 the need for more accurate and standardized delineation of endemic areas was recognized, and the mapping of disease distribution was set by WHO as a priority in its regional strategy for the control of HAT [18]. Recent and spatially-explicit epidemiological data are also needed to update the country figures of the population at risk that were last estimated in 1995 by a WHO Expert Committee [17], and which were largely based on expert opinion.

In 2007 WHO and FAO, in the framework of the Programme against African Trypanosomiasis (PAAT), joined their efforts to map sleeping sickness in sub-Saharan Africa by using, as primary source, the vast amount of epidemiological data collated by WHO in recent years. This paper describes methodology and preliminary results of this activity, as well as future prospects for the WHO/FAO initiative.

## Methods

### A geographic database of human African trypanosomiasis

**Figure 1:**
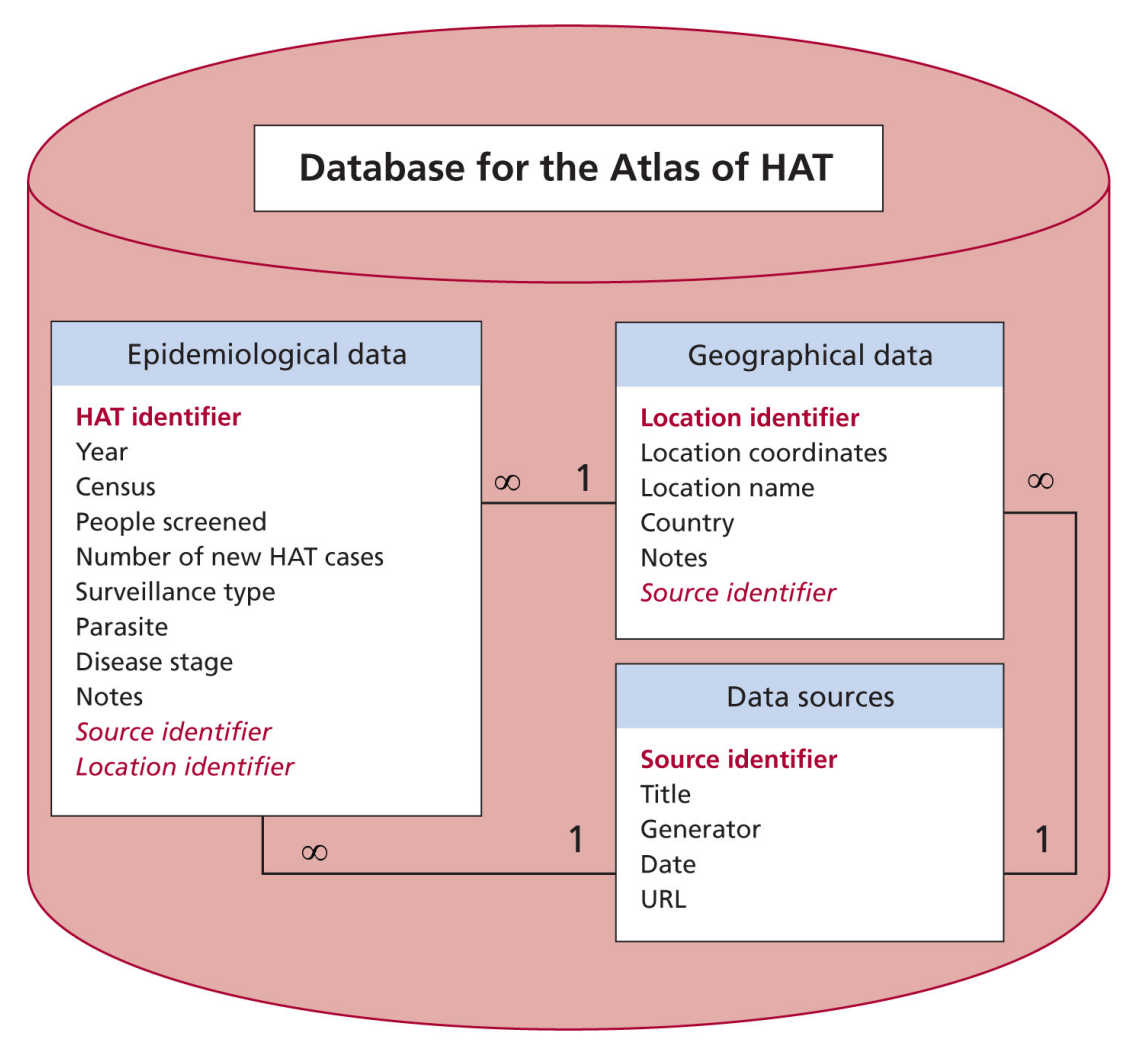
**Simplified logical model of the database of human African trypanosomiasis**. Keys are represented in red fonts (in bold are primary keys, in italics are foreign keys)

The keystone of the HAT Atlas initiative is the creation of a geographic database (DB) designed to consistently store and regularly update HAT epidemiological and spatial data. As it is demonstrated by similar regional and global disease mapping efforts [19-21], the choice of a unifying variable is the first step to map the limits and risk of disease infection. Criteria of accuracy, consistency and completeness have to be considered in order to strike a balance between the expected optimal results and the availability of input data. WHO identified five main data sources on the occurrence of human African trypanosomiasis: (i) reports of the National Sleeping Sickness Control Programmes (NSSCPs) (ii) reports of NGOs (iii) reports of research institutes (iv) data published in formal and grey literature (v) historical datasets available at WHO. After reviewing content and format of HAT records available to date, and also taking into consideration the type of information that WHO anticipates collecting in the future, the core parameters to be included in the geo-database of HAT were chosen. Figure 1 shows such core parameters as well as the DB logical model. Importantly, the number of new cases detected and people screened in a given year within a defined geographical entity were chosen as the key variables to map trypanosomiasis endemicity in sub-Saharan Africa. These variables are available in a large number of reports and they are deemed appropriate to support strategic decision-making and targeting of interventions.

The DB is composed of one section containing the core epidemiological data, another section for the description of the geographical entities the epidemiological data refer to, and a third section including the data sources used to derive both the epidemiological and the geographical information. In the epidemiological section a distinction is made between the two forms of the disease (caused by either *T.b. gambiense *or *T.b. rhodesiense*) and between the cases detected through active and passive surveillance. Disease stage, number of people screened and number of people living in the screened areas are also included when available in the reports. 'Keys' link the epidemiological records to the respective geographical entity and to data sources.

This simple structure enables to import into the HAT DB only the essential information to geo-locate and harmonize available epidemiological records, thus enabling to generate an Atlas of HAT that be consistent throughout sub-Saharan Africa. The DB structure has been kept simple and flexible so as to leave room for future expansions, which may include more detailed epidemiological information already available in many of the collected reports or to be acquired in future data collection activities (e.g. age and sex of the case detected, number of people positive to serological tests, number of relapses, etc.). The DB is envisaged to be a dynamic tool that will be regularly updated by bringing in newly generated information as it becomes available. Whenever feasible and appropriate, historical data will also be imported into the DB, with a view to providing input for longitudinal studies of longer span.

### Geo-location methodology

**Figure 2:**
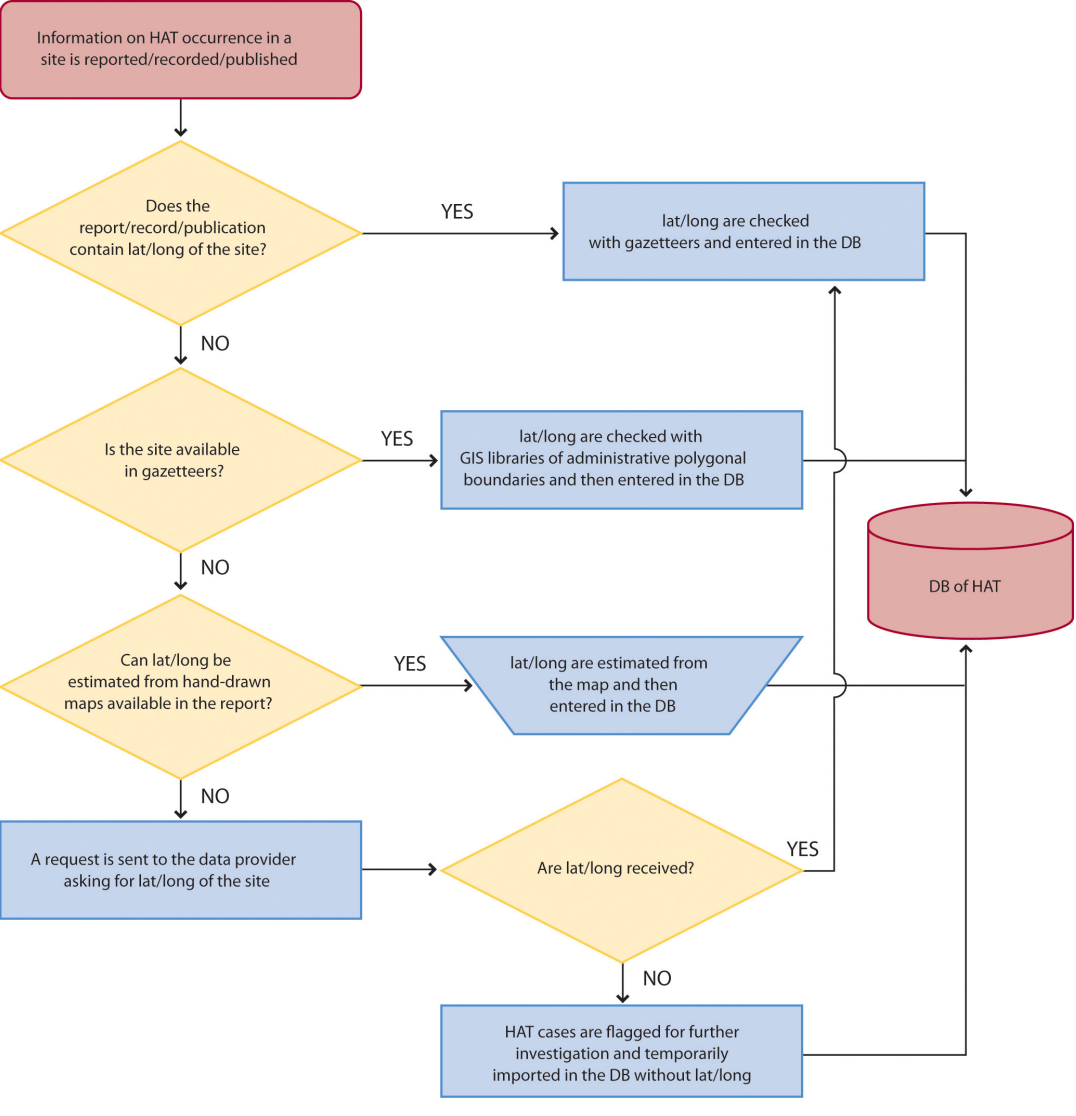
**Flow-chart for the geo-positioning of HAT cases**.

The process of assembling epidemiological data into a Geographic Information System (GIS) requires positioning the geographical entities for which disease information is available. In this context, the locations of epidemiological interest are those where active screening is carried out, as well as those locations from which HAT cases are passively reported. Figure 2 shows the flow chart of the geo-positioning process that is being used to generate the geographical section of the HAT DB.

Geographic coordinates are increasingly acquired by mobile teams that are involved in HAT active case-finding and they may also be recorded by centres belonging to networks of passive surveillance, which report the place of origin of detected cases. Geographic coordinates are normally measured by means of Global Positioning System (GPS) devices. When available in the epidemiological reports collated by WHO, geographic coordinates are stored in the HAT geo-database and used to map endemicity in the corresponding locations. If geographic coordinates are unavailable, geo-positioning of disease cases is carried out by matching the names of the locations contained in the reports with gazetteers. Gazetteers are dictionaries of geographical information that list, among other pieces of information, names and coordinates of geographical entities. Various digital gazetteers can be found on-line; as prime reference for our study we chose the GEOnet Names Server (GNS) database of the United States National Geospatial-Intelligence Agency (NGA), which provides the baseline for many, if not all, of the available gazetteers [22].

In consideration of the extensive use of GNS database in this mapping activity, its relative accuracy of 1,800 metres marks a threshold for the accuracy of the final products. Beside GNS database, other gazetteers are available which are used on an *ad hoc *basis in an attempt to solve specific geo-referencing problems (Table 1 lists the most relevant gazetteers that are available in the public domain).

**Table 1:** Public-domain databases of geo-referenced named locations. These databases are used to locate HAT cases if geographic coordinates are not available in the epidemiological report.

**Gazetteer**	**URL**
GEOnet Names Server database	
Alexandria Digital Library Gazetteer	
Getty Thesaurus of Geographic names	
Google Maps World Gazetteer – Maplandia	
Falling Rain Genomics	
Traveljournals.net	
EC Joint Research Centre Digital Atlas	

In addition to the gazetteers listed in Table 1, the present study benefits from a database of geo-referenced named locations provided by WHO's Public Health Mapping and GIS programme (PHMGP). Such a database can not be accessed on-line, even though it is available to eligible WHO partners through the HealthMapper application.

**Figure 3:**
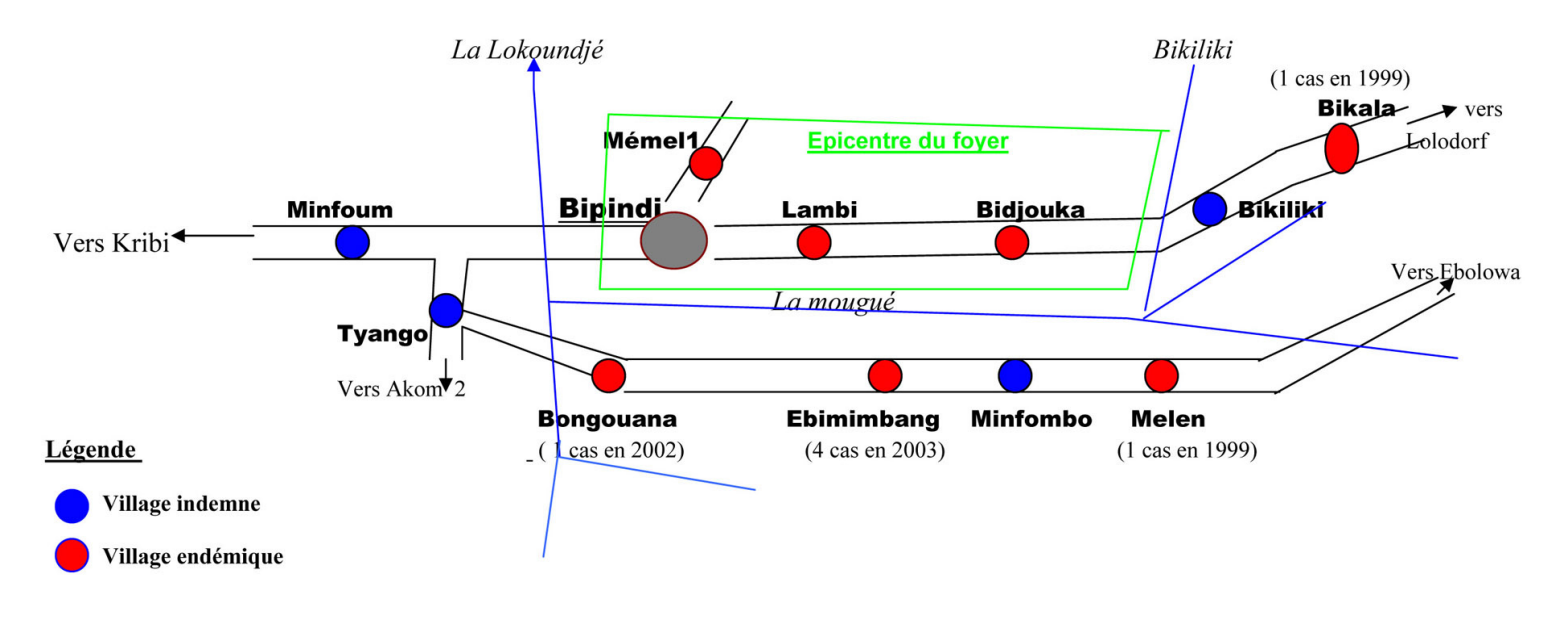
**Example of hand-drawn, out-of-scale map that may be available in HAT epidemiological reports**. This sketch map refers to the Bipindi focus in south-western Cameroon. Source [23].

In the few instances when the location of a community of unknown coordinates can not be determined with gazetteers, approximate position can often be derived from out-of-scale, hand-drawn maps that may be enclosed with or attached to reports of screening activities. One example of this type of map is shown in Figure 3[23]. Although not ideal, this method still proves effective in locating some small, otherwise uncharted villages and hamlets.

**Figure 4:**
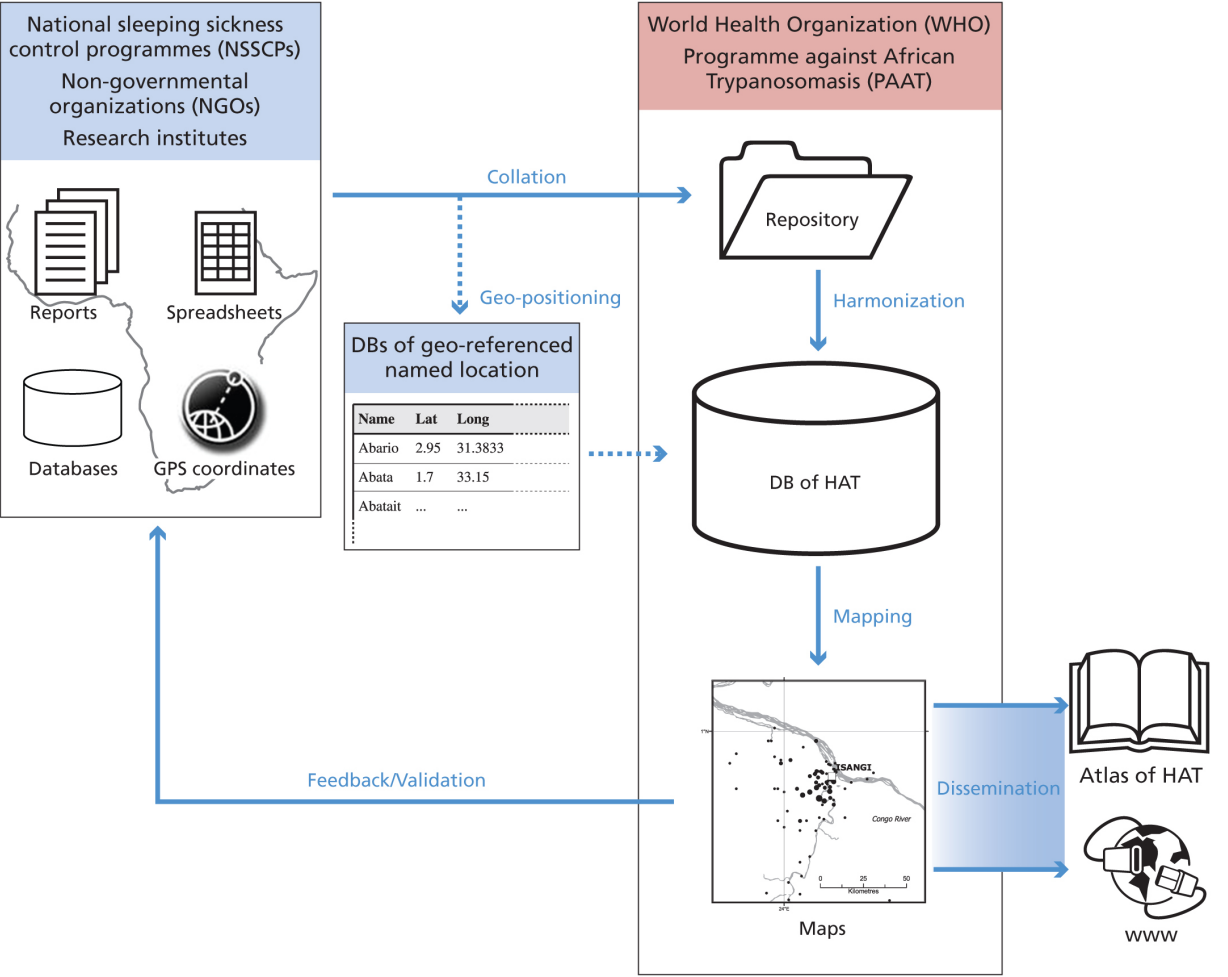
**The information system for the Atlas of HAT**. The diagram outlines data providers, input, data flow, archiving, outputs and dissemination channels of the information system of HAT.

Different methods to assure the quality of the results of the geo-location procedure were devised and put in place. If reported coordinates are used, the village names are matched with gazetteers to identify possible errors (e.g. errors in GPS readings). It should be noted here that the average accuracy of gazetteers is such that, if available and reliable, GPS coordinates ought to be preferred. When HAT cases are mapped by means of gazetteers, geo-positioning errors can be introduced by synonyms or misspelling of names; in these cases a number of the potential errors can be pinpointed by checking that the location falls in the vicinity of other villages screened during the same field mission, or that it falls within the boundaries of the appropriate administrative units. Basic GIS functions and ancillary information in surveillance reports allow these checks to be made. Lastly, WHO counterparts in the affected countries are consulted to fill gaps in the reporting of geographic coordinates. Draft epidemiological maps of HAT-affected areas are shared with NSSCPs in order to get feedback on the overall correctness of the geo-positioning procedure and with a view to consolidating the final cartographic products. This phase is also crucial to build consensus on the outcomes of the mapping activity and to create a sense of ownership in the NSSCPs, which are the primary beneficiaries of the initiative. Figure 4 synthesizes the infrastructure that is in place to map the global distribution of HAT, including formats and sources of input data, data flow and storage, outputs and dissemination channels.

## Results

At the time of writing, approximately 610 epidemiological reports and files dating from 1985 onwards were collated and included in the data repository. These reports contain information for over 20,000 HAT cases and approximately 7,000 geographical entities. Data from seventeen countries have been included in the repository so far: Angola, Benin, Burkina Faso, Cameroon, Central Africa Republic (CAR), Congo, Côte d'Ivoire, DRC, Equatorial Guinea, Gabon, Ghana, Guinea, Malawi, Mali, Sudan, Togo and Uganda.

For eleven countries, data processing has been initiated and data are being imported into the HAT database. High priority is presently given to the most recent datasets (i.e. reports dating from the year 2000 onwards).

**Figure 5:**
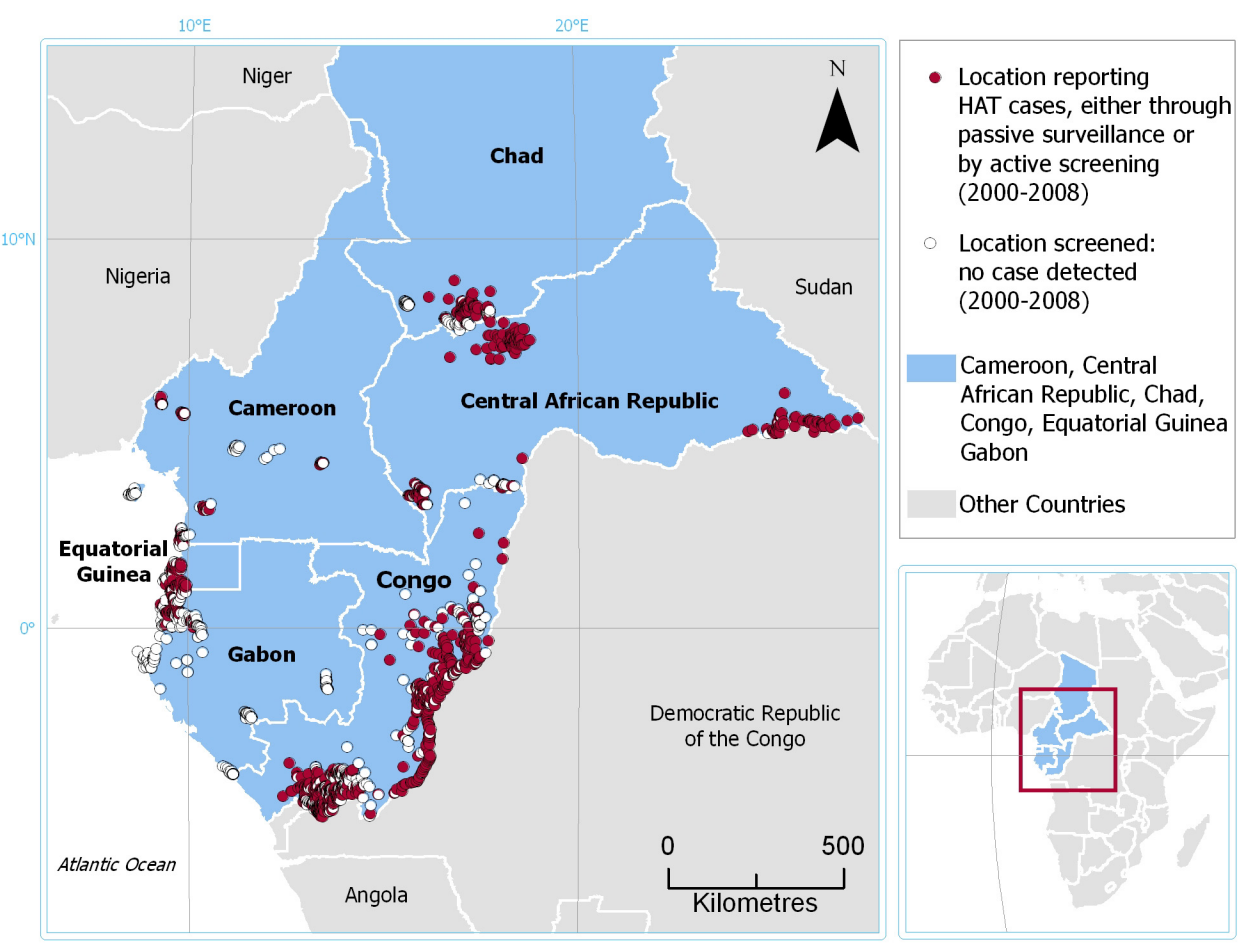
**Atlas of HAT: an example of regional-level map**. Red dots represent locations from which HAT case were reported (either through active screening or by passive surveillance); white dots indicate screened locations where no HAT case was detected. Study countries: Cameroon, CAR, Chad, Congo, Equatorial Guinea, and Gabon. Reporting period: 2000–2008. Data sources: National Sleeping Sickness Control Programmes. For CAR and Congo data from Médecins Sans Frontièrs are also included.

For six central African countries, namely Cameroon, CAR, Chad, Congo, Equatorial Guinea, and Gabon, all data available for the period 2000–2008 were processed, thus leading to the first preliminary regional component of the HAT Atlas initiative. Figure 5 shows the spatial distribution of 1,580 locations, corresponding to 92 percent of all locations of epidemiological interest included in the reports. For 829 of these locations (red dots), HAT cases were reported either by passive surveillance of through active case-finding surveys. Epidemiological data used as input for this map were collected by the NSSCPs of the six countries; for CAR and Congo, data collected by the NGO Médecins Sans Frontières (MSF) were also included.

We note that a significant number of affected locations can be found in border areas, where trade and population displacements may contribute to creating conditions conducive to disease transmission.

Table 2 provides country-level statistics on the results of the geo-referencing activity for this first regional output of the HAT Atlas initiative. Overall, the geo-referencing exercise was very successful. The locations of epidemiological interest that could be mapped as point entities (e.g. camps, hamlets, villages, towns, etc.) allowed to geo-reference 98 percent of HAT cases reported from the six study countries. In addition to villages reporting HAT cases, 751 screened locations that did not result in any positive case were also included in the DB (white dots). The importance of including these sites in the DB can not be overstated, as they play a crucial role in drawing and updating the overall epidemiological picture. In actual fact, knowing how many villages have been screened and their location is of critical importance in providing information on where there is or there is not active transmission. Thus, a database of screened but negative villages is as important as a database of screened and positive ones.

**Table 2:** Results of the geo-referencing activity in 6 central-African countries. The table shows how the geographic locations and HAT cases contained in epidemiological reports were geo-referenced with either reported coordinates, gazetteers, or other means (e.g. paper and digital maps, hand-drawn maps, etc.).

**Country**	**Geographic locations**					**HAT cases**	
	**available in the reports**	**mapped with reported coordinates**	**mapped with gazetteers**	**mapped with other resources**	**not mapped yet**	**available in the reports**	**mapped**
	[number]	[%]	[%]	[%]	[%]	[number]	[%]

Cameroon	88	51.1	30.7	14.8	3.4	141	100.0
Central Africa Republic	311	64.3	16.7	2.6	16.4	3,323	96.4
Chad	157	63.1	17.2	7.0	12.7	1,484	97.1
Congo	816	60.7	24.4	11.3	3.6	4,368	99.4
Equatorial Guinea	104	72.2	19.2	1.9	6.7	136	100.0
Gabon	233	54.6	18.4	17.6	9.4	197	97.0

TOTAL	1,709	61.5	21.8	9.0	7.7	9,649	98.0

For most geographical entities (approximately 61 percent), geographic coordinates were either available in the epidemiological reports or provided through consultation with WHO partners; 22 percent of the locations were geo-referenced using the combination of reported names and gazetteers, and for 9 percent position was estimated from other sources (digital or paper maps, out-of-scale maps enclosed with the reports, etc.). The remaining geographical entities (8 percent, associated with 2 percent of the HAT cases) have not been mapped yet.

**Figure 6:**
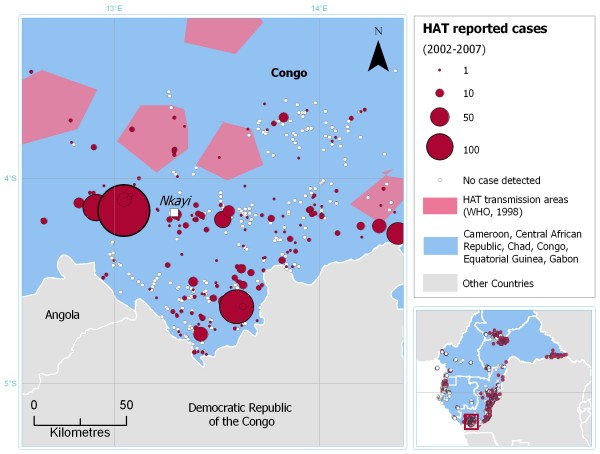
**Atlas of HAT: an example of local-level map**. Cumulative number of sleeping sickness cases recorded in the area of Nkayi, Bouenza Region (Congo), between 2002 and 2007. Data sources: Programme National de Lutte contre la Trypanosomiase Humaine Africaine (Congo) and Médecins Sans Frontières.

As an example, Figure 6 shows the cumulative number of sleeping sickness cases recorded in the area of Nkayi, Bouenza Region, Congo, between 2002 and 2007. Locations where no cases were detected, as well as the boundaries of HAT transmission area as depicted by WHO in 1998 [17] are also included.

As opposed to Figure 5, this zoomed-in image allows to fully appreciate the unprecedented spatial detail of the HAT Atlas. We note that the picture drawn in this area by the HAT Atlas appears substantially different from the previous map of HAT transmission areas. This is true also for the study region as a whole. Less than a third of the endemic locations we mapped in the six central African countries are situated within the boundaries of previously described transmission areas. Even though the spatial distribution of transmission areas may have undergone some changes in the last decade, it is believed that most of the differences between the past and present representation are to be ascribed to improved methodology for mapping control activities, rather than to a substantial evolution of the epidemiological conditions on the ground. It is important to stress that these preliminary results will be systematically verified in collaboration with NSSCPs, with a view to consolidating and sharing the outcomes of the HAT Atlas initiative.

## Discussion

The DB under development strikes a balance between what would be ideally required of a global information system for HAT and what is presently feasible, especially in the light of current data availability.

An ideal GIS on the occurrence of HAT would arguably contain detailed information on each known case of the disease, including: geographic coordinates of the patient's household, sex and age of the patient, exact date of disease detection or reporting, duration of illness, mortality/recovery rates. Also, information on the location of the patient's working place would be needed, as well as the patterns of people mobility in the area.

In practice, consistently setting up the ideal GIS of HAT in sub-Saharan Africa does not seem feasible in the short term as the available information is patchy and heterogeneous in format. For example, geographic coordinates of a patient's household and working place are hardly ever available in epidemiological reports; therefore we resorted to referring HAT cases to a different, rather less specific geographical entity, most frequently a village.

It is also necessary to underline that, in our Atlas, HAT cases either reported by networks of passive surveillance (e.g. clinics, HAT treatment centres, etc.) or detected through active screening by mobile teams are normally geo-referenced in a slightly different manner. As a general rule, the former are referred to the patient's village of residence, the latter to the place where active screening took place (which very often, albeit not always, coincides with the patient's place of residence).

Lastly, despite substantial improvements in screening and reporting activities in recent years, the problem of under-reporting continues to affect estimates of the real burden of HAT [24], as it does for many other neglected tropical diseases [25].

Notwithstanding these limitations, our preliminary results have proven how available epidemiological and geographical data are sufficiently coherent and detailed to generate a unified geo-database of human trypanosomiasis for sub-Saharan Africa.

## Conclusion

In a context of renewed commitment by international and national institutions and of subsiding conflicts in many affected countries, recent years have witnessed important progress in the control of HAT. This was achieved through more intensive and effective surveillance and reporting activities. A large amount of spatially-explicit, almost entirely unpublished epidemiological data is now available, and an effective network of NSSCPs, institutions and organizations will guarantee continuity of data collection and sharing in the future. Today it is possible to create a comprehensive, GIS-based information system for sleeping sickness at continental level, hinging on a geo-database of disease occurrence and monitoring. The system will make the production of the Atlas of HAT possible, while enabling regular updates as new data become available.

A number of applications can be envisaged for the DB and the Atlas of HAT. First and foremost is the generation of an updated map of disease transmission areas. The preliminary results for the six central African countries, here presented, revealed important differences from past disease distribution maps, thus demonstrating the inadequacy of these previous cartographic products to calculate the current population at risk. In combination with global population datasets [26] the DB of HAT will allow to devise and apply novel, evidence-based methods to estimate disease risk through GIS techniques. Importantly, the full involvement of the NSSCPs will contribute to promote and improve collection, reporting and use of geo-referenced epidemiological data, thus leading to more efficient and effective targeting of interventions. Lastly, there is a growing need to understand how environmental modifications, population dynamics and climate change will impact on the distribution and incidence of the disease. It is believed that the availability of harmonized and accurate baseline data will provide critical input to a wide range of research activities.

With falling numbers of new HAT cases detected in recent years, effective surveillance and control followed by good reporting will be vitally important to sustain the current control efforts [3]. In this context, efficient management of geospatial epidemiological information will be crucial to measure progress towards the goal of elimination of HAT as a public health problem.

## Abbreviations

Abbreviations used in the text, tables or references: CAR: Central Africa Republic; DB: Database; DRC: Democratic Republic of the Congo; FAO: Food and Agriculture Organization of the United Nations; GIS: Geographic Information System; GNS: GEOnet Names Server; GPS: Global Positioning System; HAT: human African trypanosomiasis; IFAD: International Fund for Agricultural Development; MSF: Médicins Sans Frontières; NSSCP: National Sleeping Sickness Control Programme; NGO: Non-Governmental Organization; PAAT: Programme against African Trypanosomiasis; NGA: Unites States National Geospatial-Intelligence Agency; PHMGP: WHO's Public Health Mapping and GIS programme; URL: Uniform Resource Locator; WHO: World Health Organization.

## Competing interests

The authors declare that they have no competing interests. The views expressed in this paper are those of the authors and do not necessarily reflect the views of WHO and FAO.

## Authors' contributions

GC coordinated and supervised the technical aspects related to data management and GIS and drafted the manuscript. PPS, JRF, AD and JAR collated and screened epidemiological data used as input. GC, MP, EMF and PPS designed and developed the logical and physical structure of the database of HAT. MP implemented geo-positioning procedures and entered data. EMF provided perspectives on geo-spatial analysis of HAT epidemiology. PPS and RCM coordinated and supervised the collaboration between WHO and FAO in the framework of PAAT. All authors have jointly conceptualized and contributed to the manuscript, and read and approved the final version of the submitted manuscript.

## References

[B1] Heisch RB, McMahon JP, Manson-bahr PEC (1958). The isolation of *Trypanosoma rhodesiense *from a bushbuck. Br Med J.

[B2] Onyango RJ, van Hoeve K, de Raadt P (1966). The epidemiology of *Trypanosoma rhodesiense *sleeping sickness in Alego location, Central Nyanza, Kenya. I. Evidence that cattle may act as reservoir hosts of trypanosomes infective to man. Trans R Soc Trop Med Hyg.

[B3] Simarro PP, Jannin J, Cattand P (2008). Eliminating Human African Trypanosomiasis: Where Do We Stand and What Comes Next. PLoS Med.

[B4] Welburn SC, Fèvre EM, Coleman PG, Maudlin I, Maudlin I, Holmes PH, Miles MA (2004). Epidemiology of Human African Trypanosomiasis. The Trypanosomiases.

[B5] World Health Organization (1997). Resolution 50.36, 50^th ^World Health Assembly. Geneva.

[B6] World Health Organization (2002). WHO programme to eliminate sleeping sickness – Building a global alliance. Geneva.

[B7] World Health Organization (2006). Human African trypanosomiasis (sleeping sickness): epidemiological update. Weekly epidemiological record.

[B8] Berrang Ford L (2007). Civil conflict and sleeping sickness in Africa in general and Uganda in particular. Conflict and Health.

[B9] Ebeja AK, Lutumba P, Molisho D, Kegels G, Miaka mia Bilenge C, Boelaert M (2003). La maladie du sommeil dans la région Ville de Kinshasa: une analyse rétrospective des données de surveillance sur la période 1996–2000. Trop Med Int Health.

[B10] Odiit M, Shaw A, Welburn SC, Fevre EM, Coleman PG, McDermott JJ (2004). Assessing the patterns of health-seeking behaviour and awareness among sleeping-sickness patients in eastern Uganda. Ann Trop Med Parasitol.

[B11] Berrang Ford L, Berke O, Abdelrahman L, Waltner-Toews D, McDermott J (2006). Spatial analysis of sleeping sickness in south-eastern Uganda, 1970–2003. Emerg Infect Dis.

[B12] Cattand P, Jannin J, Lucas P (2001). Sleeping sickness surveillance: an essential step towards Elimination. Trop Med Int Health.

[B13] Picozzi K, Fèvre EM, Odiit M, Carrington M, Eisler M, Maudlin I, Welburn SC (2005). Sleeping sickness in Uganda: a thin line between two fatal diseases. Brit Med J.

[B14] Leak SGA (1970). Tsetse Biology and Ecology: Their Role in the Epidemiology and Control of Trypanosomosis.

[B15] Courtin F, Jamonneau V, Duvallet G, Garcia A, Coulibaly B, Doumenge JP, Cuny G, Solano P (2008). Sleeping sickness in West Africa (1906–2006): changes in spatial repartition and lessons from the past. Trop Med Int Health.

[B16] Fèvre EM, Coleman PG, Odiit M, Magona JW, Welburn SC, Woolhouse ME (2001). The origins of a new *Trypanosoma brucei rhodesiense *sleeping sickness outbreak in eastern Uganda. Lancet.

[B17] World Health Organization (1998). Control and surveillance of African trypanosomiasis. Report of a WHO Expert Committee. World Health Organ Tech Rep Ser.

[B18] World Health Organization (2005). Control of human African trypanosomiasis: A Strategy for the African region. Geneva.

[B19] Michael E, Bundy DA, Grenfell BT (1996). Re-assessing the global prevalence and distribution of lymphatic filariasis. Parasitology.

[B20] Brooker S, Rowlandsa M, Hallera L, Savioli L, Bundya DAP (2000). Towards an atlas of human helminth infection in sub-Saharan Africa: the use of geographical information systems (GIS). Parasitol Today.

[B21] Guerra CA, Hay SI, Lucioparedes LS, Gikandi PW, Tatem AJ, Noor AM, Snow RW (2007). Assembling a global database of malaria parasite prevalence for the Malaria Atlas Project. Malar J.

[B22] Dooley JF (2005). An Inventory and Comparison of Globally Consistent Geospatial Databases and Libraries.

[B23] Ebo'o Eyenga V (2003). Situation de l'endémie sommeilleuse au Cameroun. Ministère de la Santé Publique – Programme National de Lutte contre la Trypanosomiase Humaine Africaine Yaoundé.

[B24] Fèvre EM, Wissmann BV, Welburn SC, Lutumba P (2008). The burden of human african trypanosomiasis. PLoS Negl Trop Dis.

[B25] Mathers CD, Fat DM, Inoue M, Rao C, Lopez AD (2005). Counting the dead and what they died from: an assessment of the global status of cause of death data. Bull World Health Organ.

[B26] Balk DL, Deichmann U, Yetman G, Pozzi F, Hay SI, Nelson A (2006). Determining global population distribution: Methods, applications and data. Adv Parasitol.

